# Reduced MAGI3 level by HPV18E6 contributes to Wnt/β‐catenin signaling activation and cervical cancer progression

**DOI:** 10.1002/2211-5463.13298

**Published:** 2021-10-01

**Authors:** Zhuoli Yang, Hua Liu, Ran Song, Wenxiu Lu, Haibo Wang, Siyu Gu, Xuedi Cao, Yibin Chen, Jihuan Liang, Qiong Qin, Xiaomei Yang, Duiping Feng, Junqi He

**Affiliations:** ^1^ Department of Biochemistry and Molecular Biology Beijing Key Laboratory for Tumor Invasion and Metastasis Capital Medical University Beijing China; ^2^ Department of Interventional Radiology First Hospital of Shanxi Medical University Taiyuan China

**Keywords:** cervical cancer, HPV, invasion, MAGI3, PDZ, Wnt/β‐catenin

## Abstract

Human papillomavirus type 18 (HPV18) has high carcinogenic power in invasive cervical cancer (ICC) development. However, the underlying mechanism remains elusive. The carcinogenic properties of HPV18 require the PDZ‐binding motif of its E6 oncoprotein (HPV18 E6) to degrade its target PSD95/Dlg/ZO‐1 (PDZ) proteins. In this study, we demonstrated that the PDZ protein membrane‐associated guanylate kinase, WW and PDZ domain containing 3 (MAGI3) inhibited the Wnt/β‐catenin pathway, and subsequently cervical cancer (CC) cell migration and invasion, via decreasing β‐catenin levels. By reducing MAGI3 protein levels, HPV18 E6 promoted CC cell migration and invasion through activation of Wnt/β‐catenin signaling. Furthermore, HPV18 rather than HPV16 was preferentially associated with the downregulation of MAGI3 and activation of the Wnt/β‐catenin pathway in CC. These findings shed light on the mechanism that gives HPV18 its high carcinogenic potential in CC progression.

## Graphical abstract

By reducing the level of MAGI3 protein, HPV18 E6 promotes cervical cancer cell migration and invasion through activation of Wnt/β‐catenin signaling. Furthermore, HPV18 rather than HPV16 is preferentially associated with downregulation of MAGI3 and activation of the Wnt/β‐catenin pathway in cervical cancer. These findings shed light on the mechanism that gives HPV18 its high carcinogenic potential in cervical cancer progression.

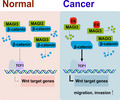

**Abbreviations**
CC: cervical cancer
CIN: cervical intraepithelial neoplasia
CIS: carcinoma *in situ*
FDR: false discovery rate
GSEA: gene set enrichment analysis
HG‐CIN: high‐grade cervical intraepithelial neoplasia
HPV16: human papillomavirus type 16
HPV18: human papillomavirus type 18
HR‐HPVs: high‐risk types of human papillomaviruses
ICC: invasive cervical cancer
IHC: immunohistochemistry
MAGI3: membrane-associated guanylate kinase, WW and PDZ domain containing 3
NHERF1: Na^+^/H^+^ exchanger regulatory factor 1
PBM: PDZ‐binding motif
PDZ: PSD95/Dlg/ZO-1
TCGA: The Cancer Genome Atlas

Cervical cancer (CC) is the fourth most prominent type of cancer in women [1]. Infection with high‐risk types of human papillomaviruses (HR‐HPVs) is the main cause for the transformation of normal cervical epithelium to cervical intraepithelial neoplasia (CIN) and progression to invasive cervical cancer (ICC) [2, 3]. The 5‐year overall survival for early‐stage patients (stage I) of CC reaches 88%, and this percentage drops to 40% for patients with more advanced disease (stages III‐IVA) [4]. The prevention including early screening and prophylactic vaccination can significantly reduce the risk of CC [5]; however, due to lack of the early prevention in some areas, especially in many developing countries, the death rate of CC is 18 times higher than that in developed countries. Vaccines have been reported to protect people from HPV infection. However, the data from the clinical trials have demonstrated that the vaccines cannot protect females already infected with HPVs against CC [6, 7]. It is estimated that there are 530,000 new cases and 270,000 deaths will occur annually worldwide [8], and the 5‐year overall survival for late‐stage CC is much lower (˜40%) [9]. Therefore, it is imperative to further explore the molecular mechanisms underlying the development and progression of ICC to provide new therapeutic options for treatment with ICC patients.

Persistent infection of high‐risk HPVs (HR‐HPVs) has been convinced to cause early carcinoma *in situ* (CIS) progression to ICC. Among HR‐HPVs, human papillomavirus type 16 (HPV16) and human papillomavirus type 18 (HPV18) are the two of the most common HPV types and cause more than 70% of ICC cases [10]. HPV16 was the most prevalent type associated with both high‐grade cervical intraepithelial neoplasia (HG‐CIN) and ICC; however, the prevalence of HPV18 was 3.5 times higher in ICC than in HG‐CIN, whereas HPV16 was only 1.1 times, indicating that HPV16 predominates in both HG‐CIN and ICC, whereas HPV18 predominates in ICC. This suggested that HPV18 rather than HPV16 promotes the progression of CINs to ICC [11]. When analyzing the age interval between HG‐CIN and ICC diagnosis, HPV18 (9 years) was significantly shorter than HPV16 (15 years) [11]. These studies suggest that HPV18 may have higher carcinogenic potential than HPV16 in CC; however, the underlying molecular mechanisms are far from clear.

So far, the viral oncoproteins E6 and E7 from HR‐HPVs have been identified as the main cancer‐causing factors of CC [12]. E7 mainly promotes the development of CC at early stages, whereas E6 plays a crucial role in the progression from in situ CC to the development of ICC [13]. Notably, the tumor suppressors of p53 and pRb are proved to be degraded or inactivated by HPV E6 and E7, respectively. The inactivation of p53 and pRb has been convinced to be mainly associated with the development of CC at early stages via promoting cell proliferation [14]. Currently, the lines of studies have shown that HPV provides the initial hit, and the activation of Wnt/β‐catenin signaling may serve as the second hit to induce malignant transformation of CC [15, 16]. Meanwhile, the PDZ‐binding motif (PBM) of E6 in HR‐HPVs is particularly important for transformation and tumorigenesis in hyperplasia and carcinogenesis in E6 transgenic mice, and transformation of primary human keratinocytes and cultured cells [17]. Minor variations in the PBM can have a dramatic effect on the interaction of PSD95/Dlg/ZO‐1 (PDZ) protein with the HPV E6 oncoproteins [18, 19], and subtle variations in the PBM region between HPV16 E6 (SSRTRRETQL) and HPV18 E6 (ERLQRRRETQV) resulted in a significant change in their binding profiles [20, 21]. Thus, it raised a possibility that HPV18 may exhibit higher carcinogenic potential through its E6‐mediated degradation of some specific PDZ proteins.

Membrane‐associated guanylate kinase, WW and PDZ domain containing 3 (MAGI3) belonging to the PDZ family of protein was more efficiently degraded by HPV18 E6 rather than HPV16 E6 [22, 23]. We previously reported that MAGI3 inhibited malignant glioma by negatively regulating the Wnt/β‐catenin signaling pathway [24]. Reports have shown that the CC progression induced by HPV18 mainly depends on activation of Wnt/β‐catenin pathway [25], whereas whether MAGI3 involved in HPV18‐associated CC is far from clear. In this study, we demonstrated that infection with HPV18 was associated with a significantly lower level of MAGI3 than paracancerous tissues; MAGI3 inhibited CC cell migration and invasion via attenuation of β‐catenin protein level and Wnt signaling. HPV18 could activate Wnt/β‐catenin signaling and promote the malignant progression of CC by reducing the amount of MAGI3 protein. Furthermore, HPV18 rather than HPV16 was preferentially associated with downregulated MAGI3 protein, and activated Wnt/β‐catenin signaling in CC. These findings shed light on the mechanism of the high carcinogenic potential of HPV18 in CC progression.

## Materials and methods

### Cell culture, reagents, and plasmids

C‐33A and HeLa cell lines were obtained from the National Infrastructure of Cell Line Resource (Beijing, China). C‐33A and HeLa cells were cultured in MEM (Corning, #10‐010‐CVR, New York, NY, USA) and DMEM (Gibco, #C11995500BT, Carlsbad, CA, USA), respectively, with 10% fetal bovine serum (FBS, Gibco) at 37 °C in an atmosphere containing 5% CO_2_. CHIR‐99021, a Wnt/β‐catenin activator, was purchased from TargetMol (Boston, MA, USA); IWR‐1‐endo, a Wnt/β‐catenin inhibitor, was purchased from Selleck (Houston, TX, USA).

pcDNA3‐V5/His‐MAGI3 and GFP/GFP‐MAGI3 plasmids were kindly provided by R. Hall (Emory University, GA). Flag‐tagged β‐catenin vector was kindly provided by W. Wu (Tsinghua University, Beijing, China). HA/Flag‐HPV18‐E6 (HA/Flag‐18E6) plasmid was kindly provided by Karl Munger (Addgene, Cambridge, MA, USA).

### Transfections and RNA interference

Cells were transfected with Lipofectamine 3000 (Invitrogen, L3000015, Carlsbad, CA, USA). The stably transfected HeLa cells were obtained as previously described [21].

The knockdown cell lines were transiently transfected with two different siRNA, which target MAGI3 and HPV18E6, and the sequences obtained as described previously [21, 24] were synthesized by Sangon Biotech (Shanghai, China) Co., Ltd. The siRNA targeting β‐catenin was purchased from Santa Cruz (sc‐29209), and the siRNA sequences obtained were as follows: β‐catenin siRNA#1: 5′‐GAUAAAGGCUACUGUUGGAUU‐3′; β‐catenin siRNA#2: 5′‐CCACUAAUGUCCAGCGUUUUU‐3′.

### Western blotting and antibodies

The western blotting assay was performed as previously described [21]. The primary antibodies used in this study were as follows: anti‐MAGI3 (Santa, sc‐136471), anti‐HPV18E6 (Santa, sc‐365089), anti‐β‐catenin (CST, #9581), and anti‐TCF1 (CST, #2206).

### Transwell migration assay and invasion assay

HeLa (5 × 10^4^) or C‐33A (5 × 10^5^) cells were suspended in 100 μL medium with 0.5% FBS and plated into Transwell chamber (Corning, #3422) that was precoated with fibronectin (Corning, #354008, 1 : 20 dilution) on the lower surface, with (migration assay) or without (invasion assay) Matrigel (Becton Dickinson Labware, #356234, 1 : 50 dilution, Bedford, MA, USA), and to the lower chamber was added 600 μL medium with 10% FBS. After culturing at 37 °C for 24 h, the cells were fixed and stained with methyl alcohol and 0.5% crystal violet, respectively. Cells were counted in 5 random fields of view per well by ImageJ, and the results were expressed as mean ± SD.

### Ethics approval

The procedures performed in this study were approved (approval no. YB M‐05‐02V.1.0) by the Ethics Committee of the Shanghai Outdo Biotech Company, and the member of the National Human Genetic Resources Sharing Service Platform (Shanghai, China, Grant no.: 2005DKA21300). The present study was performed in accordance with the ethical standards of the Institutional and National Research Committee and with the Declaration of Helsinki. Written informed consent was acquired from all patients.

### Immunohistochemistry

The human tissue microarrays containing CC and adjacent normal tissues from patients (HUteS154Su01, purchased from Shanghai Outdo Biotech Co., LTD) were analyzed by immunohistochemistry (IHC), which was performed as described previously [21]. The date of cancer grade and HPV type related to the microarray were provided by the manufacturer. Nine clinical specimens of CC with HPV18 infection were analyzed for expression of MAGI3. The tissue samples were immunostained with anti‐MAGI3 (Santa, sc‐136471). The expression score of protein was quantified by multiplying the score of staining percentage of tumor tissue (0, none; 1, 1%–25%; 2, 26%–50%; 3, 51%–75%; and 4, > 75%) and the score of staining intensity (0, no staining; 1, weak staining; 2, moderate staining; 3, strong staining; and 4, very strong staining).

### Gene set enrichment analysis

RNA‐seq data of CC samples were downloaded from The Cancer Genome Atlas (TCGA). Gene set enrichment analysis (GSEA) was performed as described (http://www.broad.mit.edu/gsea/). Predefined gene sets were obtained from the Molecular Signatures Database, MSigDB (http://software.broadinstitute.org/gsea/msigdb). False discovery rate (FDR) < 0.25 was considered statistically significant.

## Results

### HPV18 rather than HPV16 preferentially downregulates MAGI3 expression in CC specimens

**Fig. 1:**
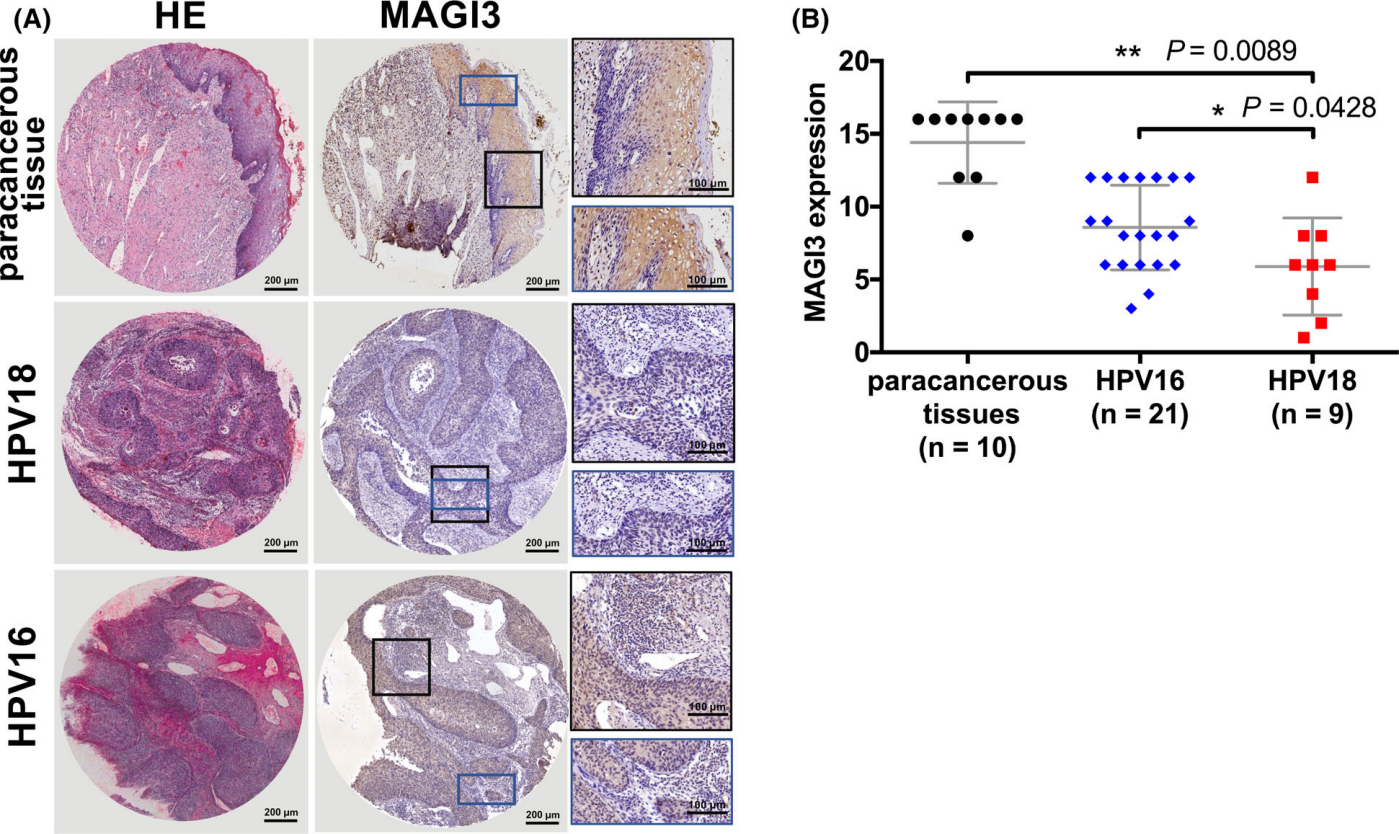
MAGI3 protein is preferentially downregulated by HPV18 rather than HPV16. (A) The expression levels of MAGI3 in microarray of adjacent cervix tissues (top panel), CC tissues of HPV18+ (middle panel), and HPV16+ (bottom panel) from clinical patients were detected by IHC staining. Scale bar, 100 μm. (B) The scatter plots of MAGI3 protein level in paracancerous vs. HPV18+ vs. HPV16+ CC tissues (nonparametric test, Mann–Whitney test, *P < 0.05, **P < 0.01, error bars represent mean ± SD).

To explore and compare the correlation of HPV18 or HPV16 infection with MAGI3 protein level in clinical CC specimens, MAGI3 was detected by IHC in a tissue microarray of CC. In HPV18‐positive CC tissues, MAGI3 level was significantly reduced when comparing to that in the adjacent noncancerous tissues or HPV16‐positive ones (Fig. 1). These findings reminded that MAGI3 might be preferentially degraded and reduced by HPV18 rather than by HPV16 in CC.

### MAGI3 inhibits migration and invasion of CC cells

**Fig. 2:**
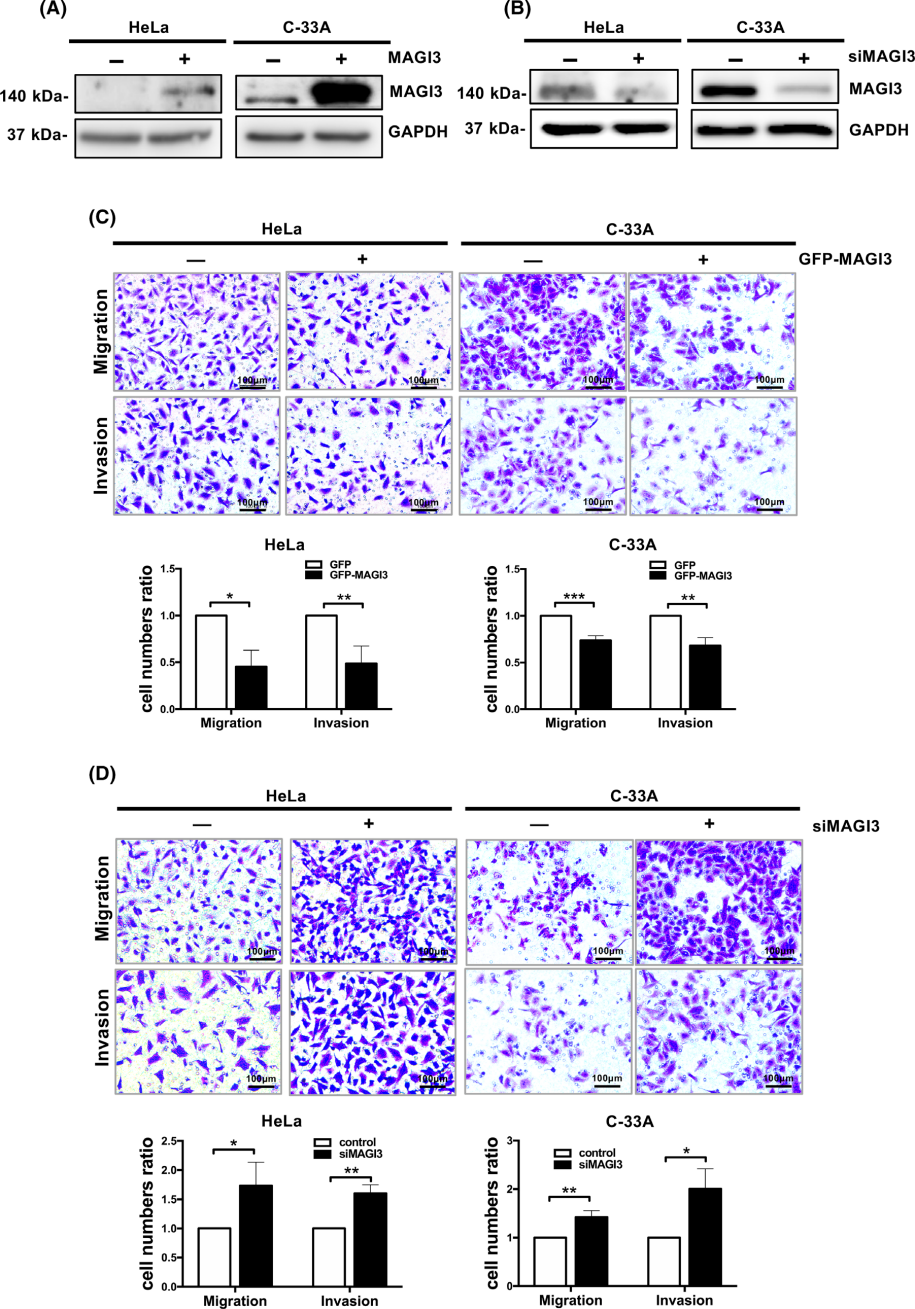
MAGI3 inhibits migration and invasion of CC cells. (A) Overexpression of MAGI3 was verified by immunoblotting. HeLa cells were stably transfected with GFP‐MAGI3 or control vector. C‐33A cells were transiently transfected with GFP‐MAGI3 or control vector. (B) MAGI3 knockdown by transient transfection with siRNAs was confirmed by immunoblotting in HeLa and C‐33A cells, respectively; GAPDH was used as a loading control. (C) MAGI3 overexpression inhibited migration and invasion of HeLa or C‐33A cells. (D) MAGI3 knockdown promoted cell migration and invasion in HeLa and C‐33A cells, respectively. Transwell migration or invasion assays were employed to test cell migration and invasion. Scale bar, 100 μm. Three independent experiments of migrating or invasive cells are represented as mean ± SD (*t*‐test; *P < 0.05, **P < 0.01, and ***P < 0.001, *n* = 3). All data were representatives of three independent experiments (*n* = 3).

Enhancement of cell migration and invasion capacity is associated with malignant progression and poor prognosis of CC [26]. To evaluate the effects of MAGI3 on migration and invasion of CC cells, MAGI3 was ectopically expressed in HeLa (HPV18 positive) and C‐33A (HPV18 negative) CC cells (Fig. 2A), the results show that overexpression of MAGI3 significantly inhibited cell migration and invasion (Fig. 2C). To verify these effects, MAGI3 was knocked down in HeLa and C‐33A cells (Fig. 2B); as anticipated, MAGI3 knockdown notably accelerated cell migration and invasion (Fig. 2D). All these data indicated that MAGI3 could inhibit the migration and invasion of CC cells.

### MAGI3 downregulates β‐catenin protein level and retards Wnt signaling in CC cells

**Fig. 3:**
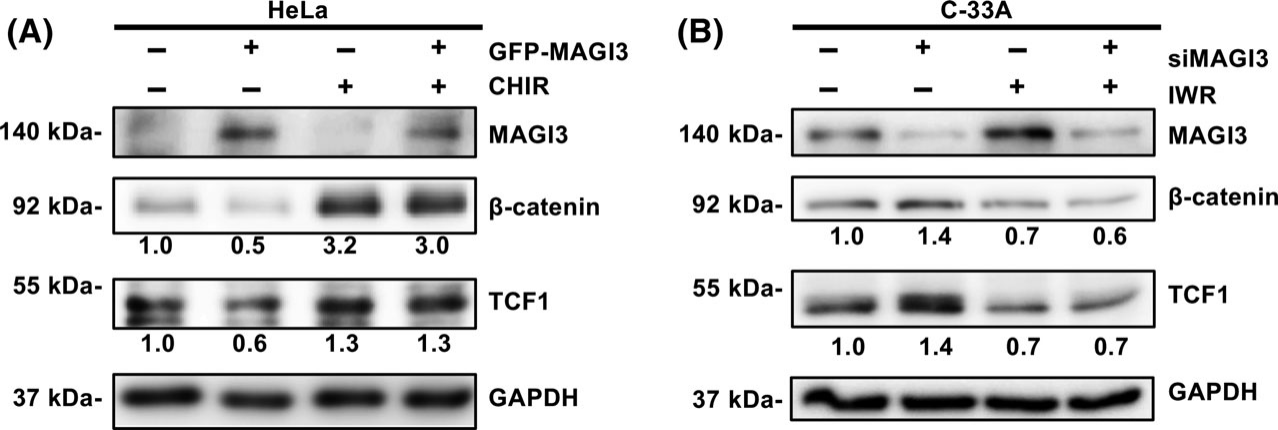
MAGI3 downregulates β‐catenin protein level and inhibits Wnt/β‐catenin‐mediated signaling in CC cells. (A) MAGI3 overexpression retarded Wnt signaling activation via reducing β‐catenin protein level. HeLa‐MAGI3 cells were treated in the presence (lanes 3 and 4) or absence (lanes 1 and 2) of Wnt/β‐catenin activator CHIR‐99021 (0.2 µmol·L^−1^) for 4 h. The expression levels of β‐catenin and TCF1 were analyzed by western blotting. (B) MAGI3 knockdown promoted Wnt signaling activation via increasing β‐catenin level. C‐33A cells were transfected with MAGI3 siRNAs or scrambles for 24 h and then treated with Wnt/β‐catenin inhibitor IWR‐1‐endo (100 µmol·L^−1^; lanes 3 and 4) for 24h, and cell lysates were analyzed by western blotting. The gray value of protein bands of western blots has been quantified by imagej. All experiments were repeated three times (*n* = 3).

Dysregulated high level of β‐catenin protein plays a crucial role in CC invasion and metastasis [15, 27, 28, 29]. We previously reported the interaction between MAGI3 and β‐catenin in glioma cells [24]. Whether and how MAGI3 can regulate β‐catenin‐mediated signaling in CC is still an open question. When MAGI3 overexpressed in HeLa cells, β‐catenin and its downstream target gene TCF1 expression levels were reduced compared with control groups (Fig. 3A, lanes 1 and 2). While cells was treated with Wnt/β‐catenin activator CHIR‐99021, the inhibitive effects of overexpression of MAGI3 on β‐catenin signaling pathway were antagonized (Fig. 3A, lanes 3 and 4). When MAGI3 was knocked down, the expression levels of β‐catenin and TCF1 were significantly increased compared with the control group (Fig. 3B, lanes 1 and 2), but the increase in β‐catenin and TCF1 was reversed by Wnt/β‐catenin inhibitor IWR‐1‐endo treatment, which downregulated the level of β‐catenin and TCF1 (Fig. 3B, lanes 3 and 4). Taken together, these data suggested that MAGI3 could inhibit β‐catenin‐mediated signaling in CC cells.

### MAGI3 retards CC cell migration and invasion through downregulation of β‐catenin

**Fig. 4:**
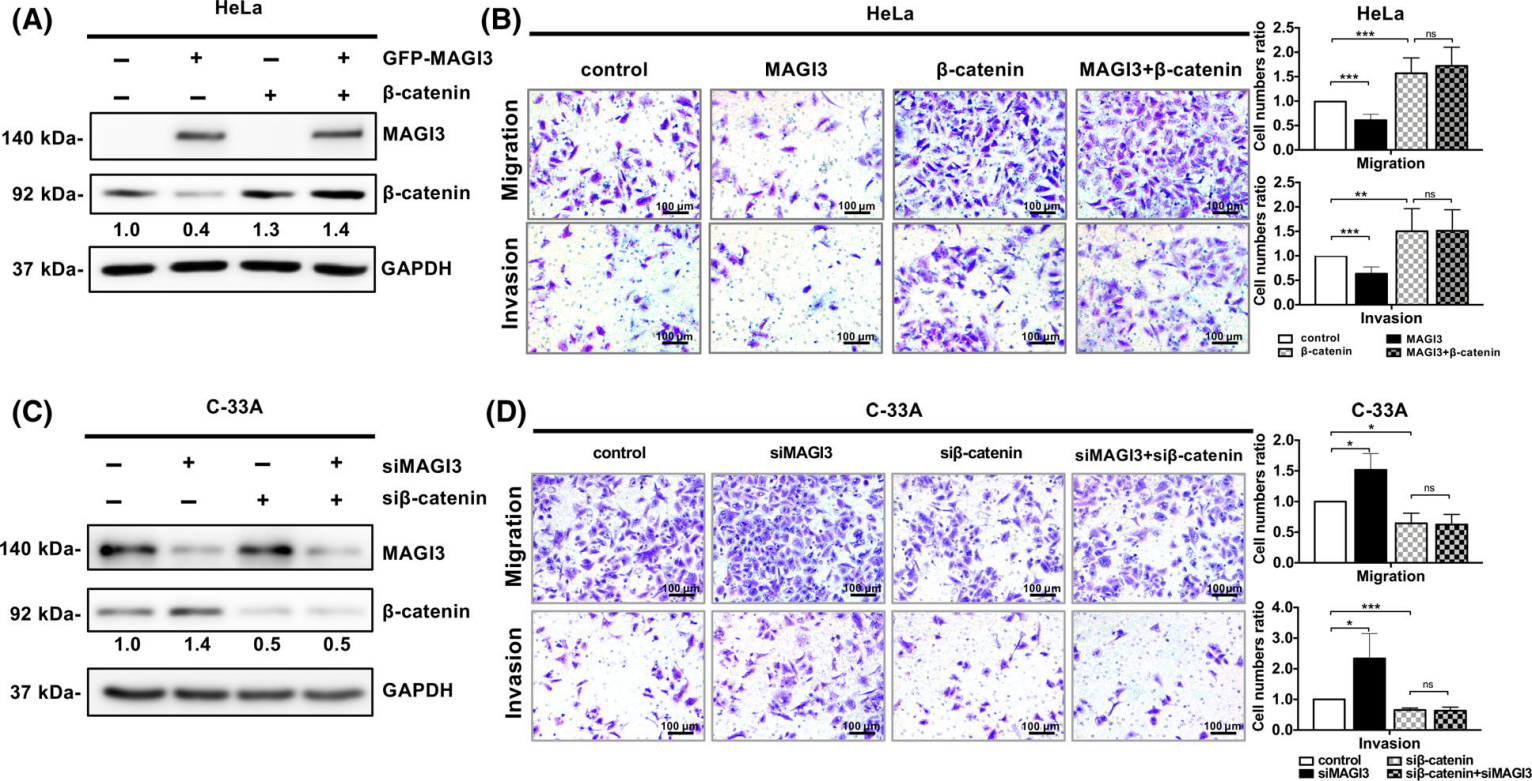
MAGI3 retards cell migration and invasion through downregulation of β‐catenin level in CC cells. (A, B) Overexpression of MAGI3 retarded cell migration and invasion via reducing β‐catenin level. The inhibition of MAGI3 on cell migration and invasion was reversed by overexpression of β‐catenin. 24 h after HeLa‐MAGI3 cells transfected with β‐catenin or vector control, the protein levels, and cell migration and invasion were analyzed as below. Scale bar, 100 μm. (C, D) Knockdown of MAGI3 in C‐33A cells significantly increased cell migration and invasion via upregulation of the protein level of β‐catenin. The cell migration and invasion were inhibited by depletion of β‐catenin in the presence or absence of MAGI3 knockdown. After being transfected with MAGI3 siRNAs with or without in combination with β‐catenin siRNAs for 24 h in C‐33A cells, cell lysates were analyzed by western blotting with indicated antibodies, and the migration and invasion were detected by Transwell assays, scale bar, 100 μm (*t*‐test; *P < 0.05, **P < 0.01, and ***P < 0.001, values represent mean ± SD, *n* = 3). The gray value of protein bands of western blots has been quantified by imagej. All data were representatives of three independent experiments (*n* = 3).

Wnt/β‐catenin signaling has been demonstrated to play an important role in regulating the migration and invasion of CC cells [30, 31],so we next investigated whether MAGI3 inhibits migration and invasion of CC cells through regulating β‐catenin expression. Overexpression of MAGI3 reduced the protein level of β‐catenin (Fig. 4A, lanes 1 and 2), and inhibited migration and invasion of HeLa cells (Fig. 4B, panels 1 and 2). When β‐catenin was rescued by ectopic overexpression (Fig. 4A, lanes 3 and 4), the inhibitive effects of MAGI3 overexpression on cell migration and invasion were reversed by the enhancement of β‐catenin protein (Fig. 4B, panels 3 and 4). Similarly, knockdown of MAGI3 increased the protein level of β‐catenin (Fig. 4C, lanes 1 and 2) and promoted migration and invasion of C‐33A cells (Fig. 4D, panels 1 and 2). MAGI3 knockdown increased β‐catenin expression (Fig. 4C, lanes 3 and 4) and enhanced cell migration and invasion; once β‐catenin expression was knocked down, the effects of MAGI3 knockdown on cell migration and invasion were reversed (Fig. 4D, panels 3 and 4). Taken together, these results indicate that MAGI3 suppressed CC cell migration and invasion through its inhibition of β‐catenin expression.

### HPV18 E6 rather than HPV16 E6 downregulates MAGI3 protein in CC cells, then leads to the activation of Wnt/β‐catenin signaling and enhancement of cell migration and invasion

**Fig. 5:**
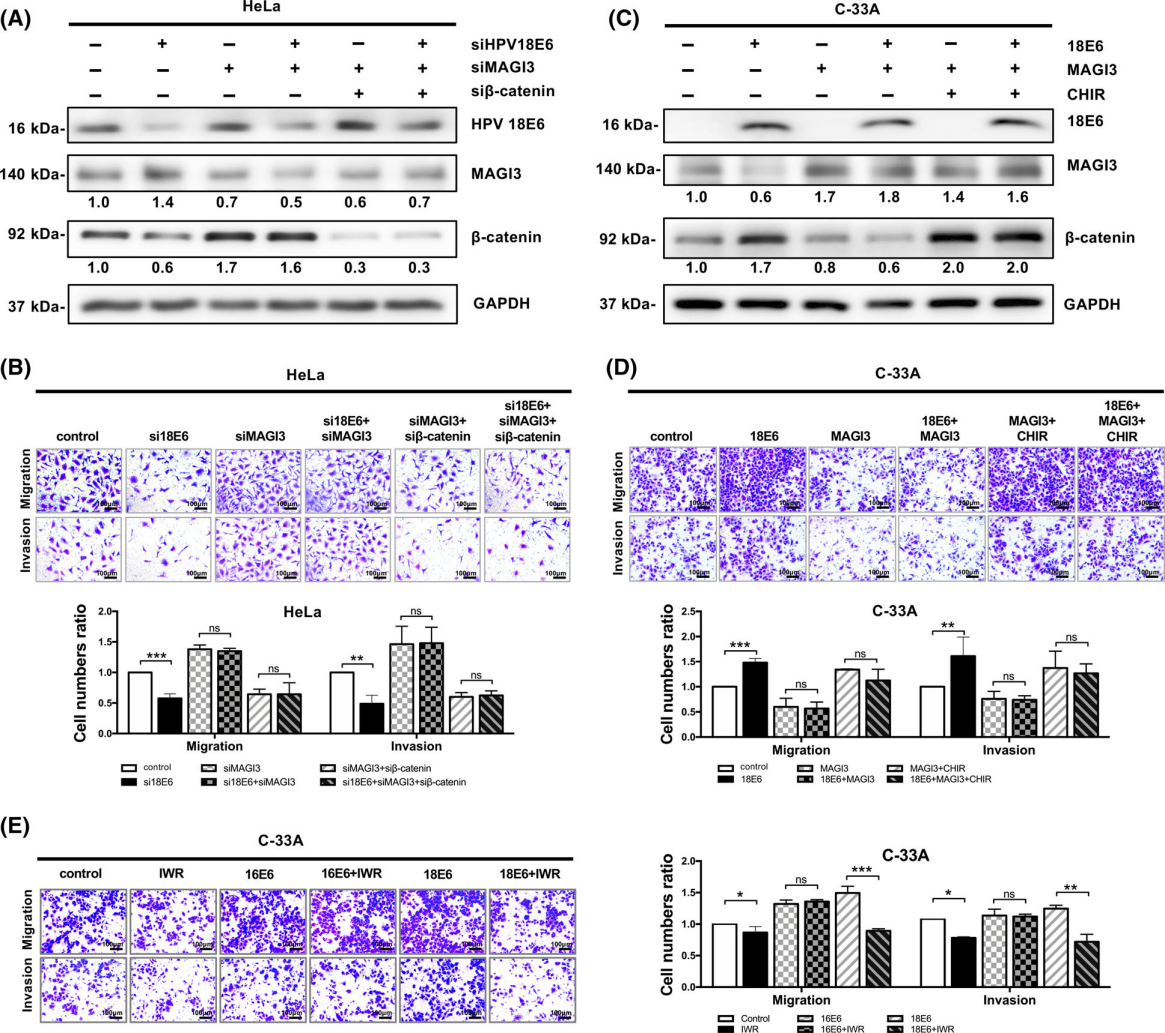
Downregulation of MAGI3 by HPV18 E6 rather than HPV16 E6 enhances cell migration and invasion via activation of β‐catenin signaling. (A, B) knockdown of HPV18 E6 inhibited the migration and invasion of HeLa cells via increasing MAGI3 and reducing β‐catenin level. HPV18 E6 was knocked down by HPV18 E6 siRNAs, or codepleted with MAGI3 siRNAs in the presence or absence of β‐catenin knockdown in HeLa cells. (C, D) Overexpression of HPV18 E6 promoted the migration and invasion of C‐33A cells via reducing MAGI3 and increasing β‐catenin level. HPV18 E6 was overexpressed alone or co‐overexpressed with MAGI3 in the presence or absence of Wnt/β‐catenin activator CHIR‐99021 in C‐33A cells. (E) HPV18 E6 rather than HPV16 E6 promoted CC cell migration and invasion through activating Wnt/β‐catenin signaling. HPV18 E6 and HPV16 E6 were overexpressed in C‐33A cells, or co‐treated with Wnt/β‐catenin inhibitor IWR‐1‐endo or vehicle. Cell lysates, migration and invasion were conducted as described in Fig. 4. The expression level of protein was quantitatively expressed by relative gray value, all experiments were repeated three times (*t‐*test; *P < 0.05, **P < 0.01, and ***P < 0.001, values represent mean ± SD, *n* = 3). Scale bar, 100 μm.

MAGI3 protein level in HPV18+ cancerous tissues is significantly lower than that in normal tissues (Fig 1), whereas MAGI3 mRNAs levels are similar between them in clinical specimens (Fig. S1). It has been reported that MAGI3 protein can be degraded by HPV18 E6 [22, 23, 32], and HPV18 E6 induced CC progression by activating Wnt/β‐catenin signaling, so we asked whether HPV18 E6 can activate Wnt/β‐catenin signaling to promote CC cell migration and invasion through degrading MAGI3 protein. While endogenous HPV18 E6 was knocked down in HeLa cells (HPV18 positive), an increased protein level of MAGI3 and decreased protein expression of β‐catenin were observed (Fig. 5A, lanes 1 and 2); meanwhile, an obvious suppression of cell migration and invasion was observed upon depletion of HPV18 E6 (Fig. 5B, panels 1 and 2). While MAGI3 was knocked down, the elevated β‐catenin level was observed whether in the presence or absence of HPV18 E6 expression (Fig. 5A, panels 3 and 4), and the CC cell migration and invasion were enhanced (Fig. 5B, panels 3 and 4). Once β‐catenin level was reduced by knockdown with siβ‐catenin, the CC cell migration and invasion were also inhibited, either with knockdown of MAGI3 or in combination with depletion of MAGI3 and HPV18 E6 (Fig. 5B, panels 5 and 6). Consistently, upon ectopic expression of HPV18 E6 in C‐33A (HPV18‐negative) to simulate CC cell infected by HPV18, downregulated MAGI3 protein expression and elevated β‐catenin level (Fig. 5C, lanes 1 and 2), and an enhanced cell migration and invasion (Fig. 5D, panel 1&2) were observed. When MAGI3 was ectopically overexpressed to reverse the downregulation of MAGI3 by HPV18 E6, β‐catenin protein level was reduced (Fig. 5C, lanes 3 and 4), and HPV18 E6 overexpression‐induced enhancement of cell migration and invasion was reversed (Fig. 5D, panels 3 and 4). Furthermore, when C‐33A cells were treated with Wnt/β‐catenin activator CHIR‐99021 to upregulate β‐catenin expression in similar levels (Fig. 5C, lanes 5 and 6), the cell migration and invasion of C‐33A cells were also similar in the presence or absence of MAGI3 / HPV18 E6 overexpression (Fig. 5D, panels 5 and 6). Taken together, these data indicated that HPV18 E6 promotes CC cell migration and invasion through inhibiting MAGI3 expression and then activating β‐catenin signaling.

In order to compare the roles of HPV18 E6 and HPV16 E6 on MAGI3 levels, Wnt/β‐catenin signaling, and cell migration and invasion, HPV18 E6 and HPV16 E6 were overexpressed in C‐33A cells. As expected, HPV18 E6 significantly reduced MAGI3 expression level and increased β‐catenin, while HPV16 E6 overexpression had no obvious reduction of MAGI3 level, but a slight increase in β‐catenin was found (data not shown). Consequently, both HPV18 E6 and HPV16 E6 promoted cell migration and invasion of C‐33A cells (Fig. 5E, panels 3 and 5). When treated with Wnt/β‐catenin inhibitor IWR‐1‐endo, the cell migration and invasion of C‐33A cells were robustly inhibited in HPV18 E6‐ but not in HPV16 E6‐overexpressed cells (Fig. 5E, panels 4 and 6), suggesting HPV16 E6‐induced cell migration and invasion of C‐33A cells were not mainly through activation of Wnt/β‐catenin signaling. Take together; these results indicate HPV18 E6 rather than HPV16 E6 promoted CC cell migration and invasion through reducing MAGI3 level and activating Wnt/β‐catenin signaling.

### Infection with HPV18 rather than HPV16 is significantly associated with the activation of Wnt/β‐catenin signaling in CC specimens

**Fig. 6:**
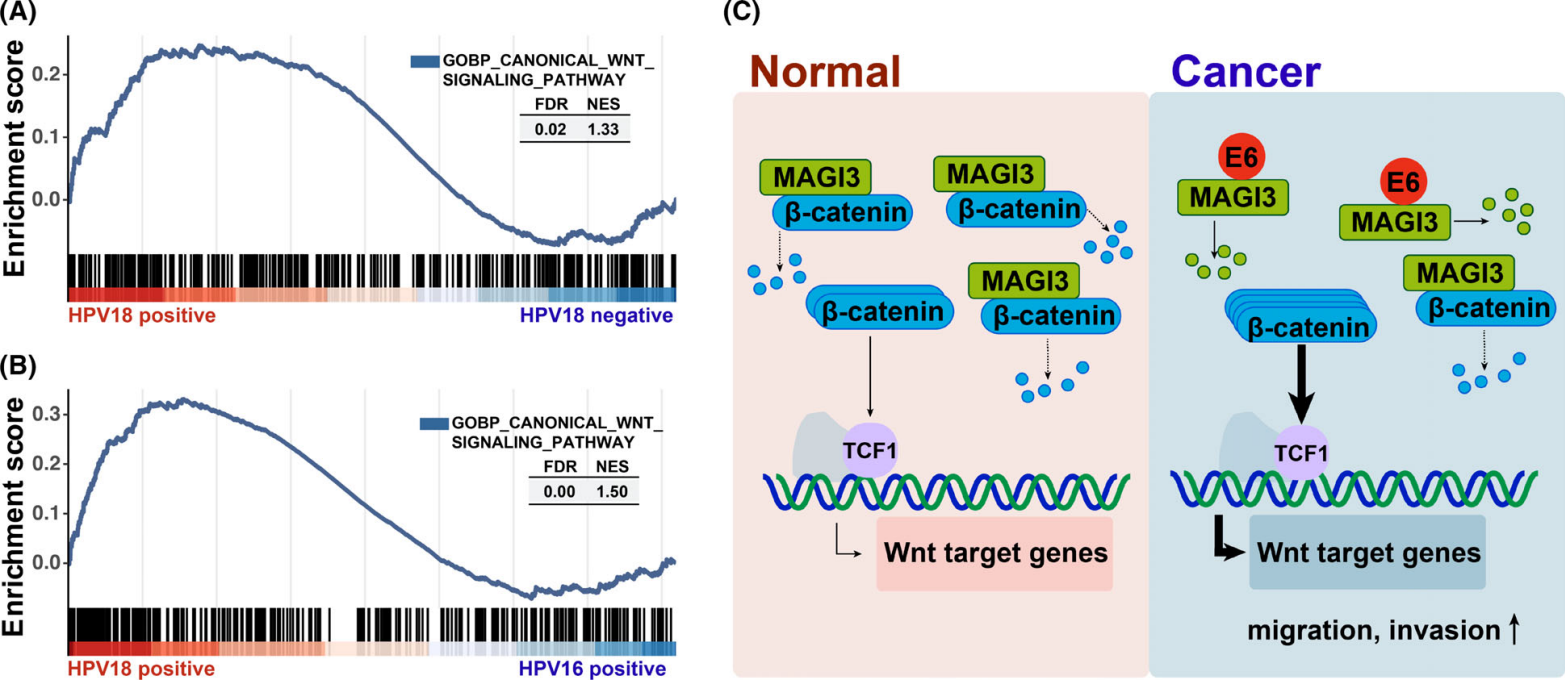
HPV18 rather than HPV16 is significantly associated with activation of Wnt/β‐catenin signaling in CC specimens. (A) Wnt/β‐catenin signaling activation is positively correlated with HPV18 infection in CC specimens. The gene signatures of Wnt/β‐catenin signaling activation were enriched in the groups of patients with HPV18 infection. The CC samples of HPV18 positive and HPV18 negative were collected from TCGA database, and Wnt pathway activation was analyzed by GSEA. (B) The gene signatures of Wnt/β‐catenin signaling activation were enriched in the HPV18 E6+ group than that in the HPV16 E6+ group; the CC data (< 43.5 years old, median age) from TCGA database were analyzed by GSEA. (C) Schematic illustration of the mechanism of CC development due to HPV18 infection via downregulation of MAGI3 and activation of Wnt/β‐catenin signaling.

We have demonstrated that MAGI3 was significantly downregulated in HPV18‐positive specimen when comparing to the adjacent noncancerous tissues or the HPV16‐positive ones (Fig. 1). To further analyze the correlation between HPV18 infection and Wnt/β‐catenin signaling activation in clinical specimen, the CC samples from TCGA database were analyzed by GSEA, as shown in Fig. 6A, and the gene signatures of Wnt/β‐catenin signaling activation were enriched in the groups of patients with HPV18 infection. These results remind that low level of MAGI3 by HPV18 infection and degradation activated Wnt/β‐catenin signaling and promoted CC progression (Fig. 6C).

GSEA by using CC data from TCGA database showed that the gene signatures of Wnt/β‐catenin signaling activation was enriched in the HPV18 E6‐positive group than that in the HPV16 E6‐positive group (Fig. 6B), suggesting that HPV18 may play more important roles to activate Wnt/β‐catenin signaling in CC development than HPV16.

## Discussion

High‐risk HPV infection is the main cause of invasion for CC. One of the most important mechanisms is that HR‐HPV E6 oncoprotein can specifically bind and degrade its target PDZ proteins through the interaction mediated by PBM at the c terminus of HR‐HPVs E6; mutation of this PBM in E6 significantly impairs its transforming activity and its ability to induce the development of CC [20]. In this study, we found that HPV18 E6 could reduce MAGI3 expression at protein level, and the loss of MAGI3 protein promotes the migration and invasion of CC cells by enhancing β‐catenin protein level and activation of Wnt signaling.

MAGI3 characterized by a prominent structural feature contains five PDZ domains. It can bind its target proteins via PBM to form macromolecular complexes to regulate the function of its target proteins and their related signal pathways [33, 34]. We previously showed that MAGI3 inhibited malignant phenotypes of glioma through negatively regulating Wnt pathway by inhibiting β‐catenin transcriptional activity [24]. However, the mechanism by how MAGI3 regulates Wnt/β‐catenin signaling and the role of MAGI3 in CC are largely unknown. In the present study, we provided evidences that MAGI3 decreased β‐catenin protein expression and retarded Wnt/β‐catenin signaling pathway to suppress migration and invasion of CC cells. This conclusion was drawn on either overexpression or knockdown of MAGI3 to inhibit or enhance the invasion ability of CC cells (Fig. 2), whereas the effects of overexpression or knockdown of MAGI3 were diminished when β‐catenin protein expression was rescued by ectopic expression or depletion of β‐catenin (Fig. 4). All the findings demonstrated that MAGI3 inhibits CC cell migration and invasion by reducing β‐catenin protein level; however, the precise molecular mechanism by how MAGI3 decreases β‐catenin protein level still needs further investigation.

HPV18 E6 was found to induce Wnt/β‐catenin pathway activation and promote CC progression [35]; HPV18 E6 was also found to target MAGI3 for its degradation [32]. In this study, we proved that MAGI3 plays a crucial role in inhibiting Wnt/β‐catenin signaling activation by reducing β‐catenin protein level in CC cells (Fig. 2), and HPV18 E6 reduces MAGI3 level leading to activation of Wnt signaling and enhancement of migration and invasion in CC cells (Fig. 5). Therefore, it is possible that low level of MAGI3 reduced by HPV18 E6 contributes to activation of Wnt/β‐catenin signaling pathway, which consequently results in the development of ICC. As expected, in clinical specimens, we observed the positive correlation of HPV18 infection with the downregulated MAGI3 protein level and the activation of Wnt/β‐catenin signaling (Fig. 6). Taken together, these results indicate that loss of MAGI3 was closely associated with the CC development; and HPV18 infection could cause development of CC via reduced MAGI3 protein level.

HPV18 has shown a higher carcinogenic potential than HPV16 in development of ICC [11, 36, 37]. The poorly differentiated CC was more frequent in HPV18 than that in HPV16‐infected cases [38]. In our previous study, HPV16 but not HPV18 was found to promote CC progression via downregulation of PDZ protein Na^+^/H^+^ exchanger regulatory factor 1 (NHERF1), which attenuated α‐actinin‐4 expression [27] since the expression level of NHERF1 was dramatically decreased by HPV16 E6 rather than by HPV18 E6 [21]. Here, we proved that reduced MAGI3 protein level by HPV18 E6 but not by HPV16 E6 contributes to Wnt/β‐catenin signaling activation (Fig. 6) and CC malignant progression (Fig. 5E).

Altogether, our results provide a mechanism by which HPV18 E6 promotes malignant progression of CC through reducing MAGI3 protein level, leading to Wnt/β‐catenin signaling activation. It provides promising clinical strategies for treatment with HPV18‐positive patients of CIN and ICC through the application of Wnt/β‐catenin signaling pathway inhibitors, which has been applied to phase II clinical trial [29, 39], or in combination with proteasome inhibitors for blocking HPV18‐mediated MAGI3 degradation.

## Conflict of interest

The authors declare no conflict of interest.

## Author contributions

JH, HL, RS, and ZY designed the experiments and analyzed data. HL, ZY, and JH prepared the manuscript. ZY and WL performed the experiments. DF contributed to sample collection, data analysis, and review of the manuscript. HW, SG, XC, YC, and JL contributed to bioinformatics and statistical analysis and critically revised the manuscript. QQ and XY analyzed data, and reviewed and revised the manuscript. JH conceived and supervised the project.

## Supporting information


**Fig. S1**. No statistical difference of MAGI3 mRNAs levels between HPV18+ vs. normal or HPV16+ tissues. The Scatter plots of MAGI3 mRNA level in normal, HPV18+ and HPV16+ CC clinical specimens from TCGA (nonparametric test, Mann–Whitney test; ns, no significance, values represent mean ± SD)

## Data Availability

The data in this study are available from the corresponding author upon reasonable request.
