# Supplementary figures and images for: Reduced MAGI3 level by HPV18E6 contributes to Wnt/β‐catenin signaling activation and cervical cancer progression
Source: FEBS Open Bio. 2021 Oct 1;11(11):3051–62. doi: 10.1002/2211-5463.13298 (PMC8564337; doi:10.1002/2211-5463.13298)

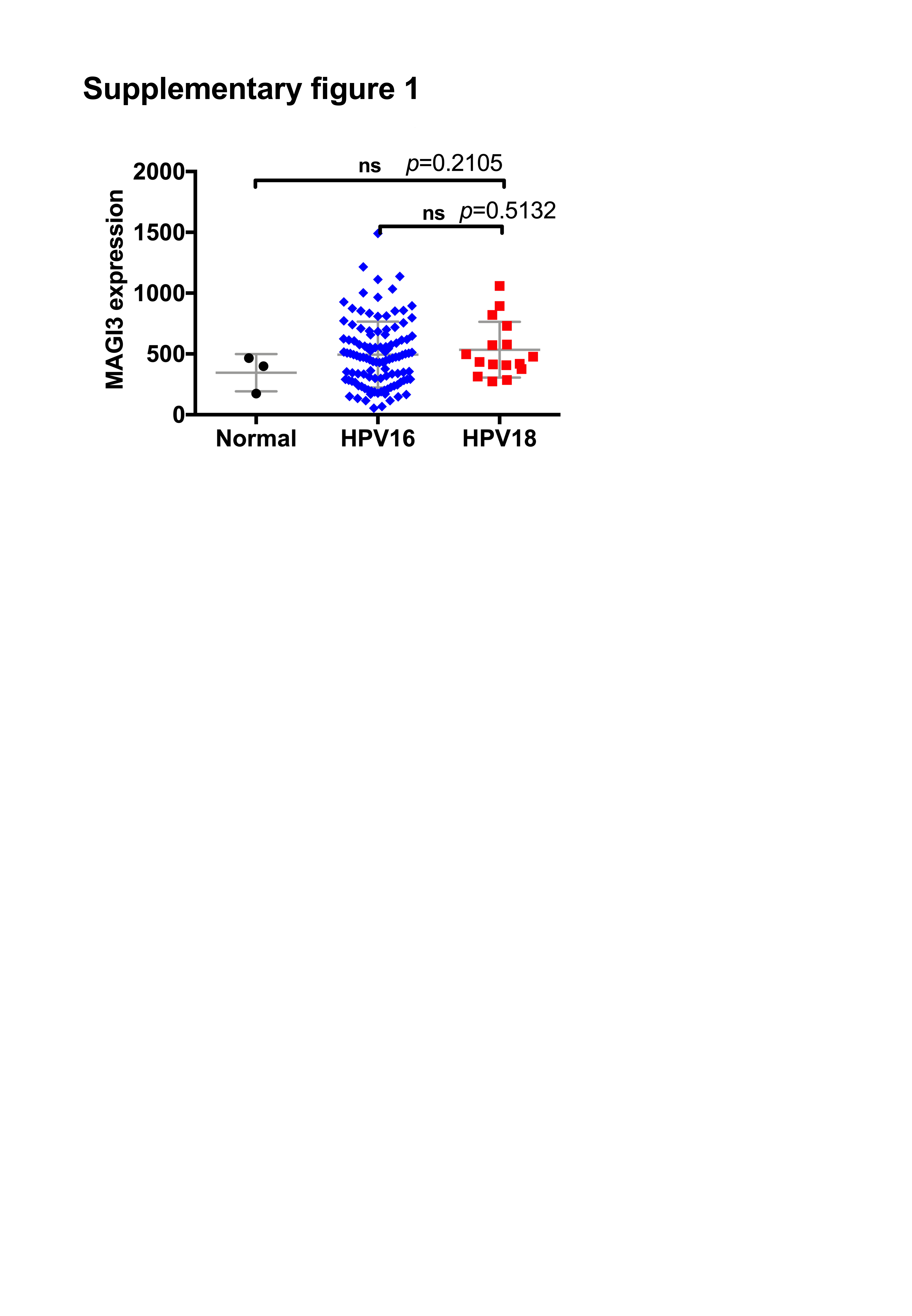

Supplement: Supplementary file 1 — Fig. S1. No statistical difference of MAGI3 mRNAs levels between HPV18+ vs. normal or HPV16+ tissues. The Scatter plots of MAGI3 mRNA level in normal, HPV18+ and HPV16+ CC clinical specimens from TCGA (nonparametric test, Mann–Whitney test; ns, no significance, values represent mean ± SD) [file FEB4-11-3051-s001.tif]
